# The presence of extracellular microRNAs in the media of cultured *Drosophila* cells

**DOI:** 10.1038/s41598-018-35531-z

**Published:** 2018-11-23

**Authors:** Stijn Van den Brande, Marijke Gijbels, Niels Wynant, Dulce Santos, Lina Mingels, Yannick Gansemans, Filip Van Nieuwerburgh, Jozef Vanden Broeck

**Affiliations:** 1 Research group of Molecular Developmental Physiology and Signal Transduction, KU Leuven, Zoological Institute, Naamsestraat 59, 3000 Leuven, Belgium; 2 Laboratory of Pharmaceutical Biotechnology, Faculty of Pharmaceutical Sciences, Ghent University, Ottergemsesteenweg 460, 9000 Ghent, Belgium

## Abstract

While regulatory RNA pathways, such as RNAi, have commonly been described at an intracellular level, studies investigating extracellular RNA species in insects are lacking. In the present study, we demonstrate the presence of extracellular microRNAs (miRNAs) in the cell-free conditioned media of two *Drosophila* cell lines. More specifically, by means of quantitative real-time PCR (qRT-PCR), we analysed the presence of twelve miRNAs in extracellular vesicles (EVs) and in extracellular Argonaute-1 containing immunoprecipitates, obtained from the cell-free conditioned media of S2 and Cl.8 cell cultures. Next-generation RNA-sequencing data confirmed our qRT-PCR results and provided evidence for selective miRNA secretion in EVs. To our knowledge, this is the first time that miRNAs have been identified in the extracellular medium of cultured cells derived from insects, the most speciose group of animals.

## Introduction

Non-coding small RNAs play a significant role in regulating gene expression in various organisms. One main class of these are the microRNAs (miRNAs) which were first reported in *Caenorhabditis elegans* to regulate timing of development^1^. Since then, thousands of new miRNAs have been discovered in a wide range of metazoan species, with key roles in presumably every biological/physiological process^2–6^. Until now, a substantial number of insect miRNAs has been recorded in miRBase (Release 21.0): more than 3000 miRNAs are already identified in 26 insect species^7^.

MiRNAs are small, *ca*. 21 nucleotides (nt) long, non-coding RNA molecules playing a crucial role in the post-transcriptional regulation of gene expression. The miRNA biogenesis pathway in insects starts in the nucleus and consists of several steps. In brief, RNA polymerase II mediates the transcription of miRNA loci^8^. The resulting transcripts fold into a hair-loop structure, called the primary miRNA, which is processed in the nucleus by the Drosha/Pasha microprocessor complex to form a precursor miRNA (pre-miRNA) of approximately 70 nt^9,10^. Subsequently, this pre-miRNA is transported into the cytoplasm by RanGTP/Exp-5^11^, where the ribonuclease III Dicer-1^12^ processes it into a miRNA duplex. This duplex is loaded into an RNA induced silencing complex (RISC) where strand selection occurs^13^. The miRNA guide strand targets messenger RNA (mRNA) via complementary base pairing, whereas the passenger strand is degraded^14^. The resulting miRNA-RISC regulates the expression levels of cognate mRNAs either by translational blockage or by cleavage via its catalytic component, an Argonaute (Ago) protein^13^. This regulation is highly dependent on the level of sequence complementarity between the miRNA and the target sequence, especially in the seed region, a contiguous string of at least six nt starting from position two of the miRNA’s 5′ end^15^. In insects, miRNAs associate with Ago-1 to mediate gene regulation^13^ while siRNAs interact with Ago-2 for antiviral immunity^16^. Mammals, however, obtained an adaptive immune response through evolution and seem to not rely on siRNA-mediated antiviral immunity; hence, mammalian Ago-2 mainly associates with miRNAs^17^.

Even though regulatory RNA pathways are traditionally described at an intracellular level, miRNA molecules have also been observed in the biological fluids of some plants and animals^18,19^. In recent years, these extracellular miRNAs (ex-miRNAs) were extensively studied in mammalian systems due to their potential as biomarkers for several life threatening conditions^20^. In this context, many studies report on the presence of specific circulating miRNA repertoires in several types of cancer, cardiovascular diseases, viral infections, as well as in brain and liver injury^20–23^.

Given that extracellular fluids contain high levels of RNase activity, the stability of ex-miRNAs in blood plasma was investigated. Incubation of synthetic miRNAs in human plasma resulted in their rapid degradation, while endogenous plasma miRNAs remained stable, suggesting that they are protected against plasma RNase activity^24^. Moreover, circulating ex-miRNAs appeared to be extremely stable as they can survive multiple freeze-thaw cycles, extreme variations in pH, boiling and extended storage^23,25,26^. Although the mechanism for this remarkable stability was initially unclear, it has been observed that many miRNAs co-purify with exosomes and microvesicles exported by cultured mammalian cells. This raised the hypothesis that ex-miRNAs are protected by encapsulation in extracellular vesicles (EVs)^27,28^. Likewise, miRNAs have been observed in EVs from human blood and basically all other extracellular fluids, such as semen, urine, cerebrospinal fluid, saliva and breast milk^25,29–35^.

Nevertheless, the assumption that only EV-encapsulated miRNAs are present in biological fluids was challenged by the observation that 95–99% of ex-miRNAs are EV-free but rather bound to proteins of the Ago family in mammalian plasma, serum and cell culture media^28,36^. In addition, the fact that mature miRNAs are associated with the (remarkably stable) Ago proteins to mediate RNAi intracellularly can explain the persistence of Ago-associated miRNAs in harsh extracellular environments^28^.

In this context, progress has been made regarding the role of ex-miRNAs in mammalian fluids. Multiple studies describe the absence of numerous miRNAs in human plasma and conditioned media, although the miRNAs are present intracellularly, suggesting controlled secretion^23,37^. Moreover, the functional transfer of exosomal mRNA and miRNA to recipient mammalian cells has also been demonstrated^27,38^. Taken together, these observations point towards the possible role of ex-miRNAs in intercellular communication.

Interestingly, in the honey bee, *Apis mellifera*, it has been demonstrated that ex-miRNAs in worker and royal jellies can regulate caste determination^39^. However, in insects, no other studies reporting on the presence of ex-miRNAs have been published. In particular, the presence of miRNAs in cell-free culture media of insect cells has not been assessed as yet. Therefore, we investigated the presence of ex-miRNAs in EVs and extracellular Ago-1 containing immunoprecipitates (EAgo-1) in the conditioned cell-free media of two cultured *Drosophila* cell lines. Here, we show that ex-miRNAs are indeed present in EVs and EAgo-1 derived from the extracellular media of cultured Cl.8 and S2 cells. Moreover, observations based on the comparison of cellular miRNA expression levels and their respective abundances in EVs point towards the selective miRNA sorting and secretion.

## Results

### Detection of miRNAs in extracellular vesicles and bound to extracellular Argonaute-1

To investigate the presence of ex-miRNAs, the well-known *Drosophila* S2 and Cl.8 cell lines were cultured. These cell lines were selected based on their distinct origin and different sensitivity for exogenous dsRNA. Haemocyte-like S2 cells were derived from late stage embryos and perform exogenous dsRNA uptake to effectuate a systemic RNAi response^40,41^. Cl.8 cells were obtained from wing imaginal discs and are characterized for their inability to take-up exogenous dsRNA, but rather require transfection to present a potent silencing response^42,43^. Based on both distinct features, a differential analysis of the ex-miRNAs of both cell lines was performed to study commonalities and discrepancies.

**Figure 1:**
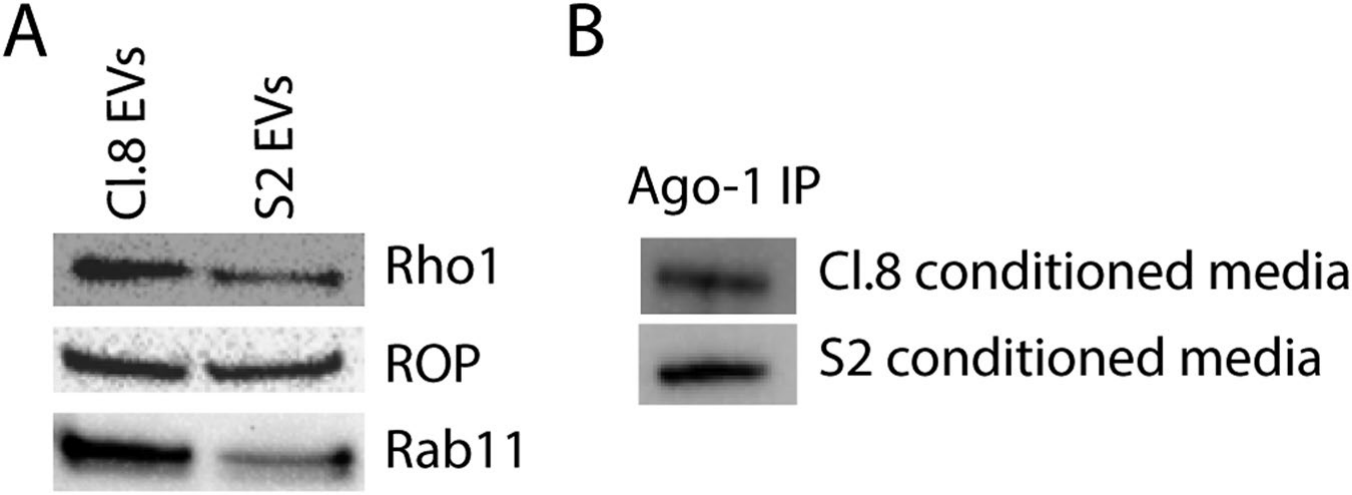
Immunoblot analysis on EVs and EAgo-1 immunoprecipitates. (**A**) EVs of both cell lines were subjected to immunoblot analysis for three common EV markers, which were clearly detected in the EV fractions purified from the extracellular media of both cell lines. (**B**) EAgo-1 IP was performed on the conditioned S2 and Cl.8 media as described in Methods section, but eluted in lithium dodecyl sulfate (LDS) instead of Qiazol. After elution, S2 and Cl.8 IP products were subjected to immunoblot analysis for EAgo-1, which was clearly detected in both IP products. Full-length blots are presented in Supplementary Fig. S1.

**Figure 2:**
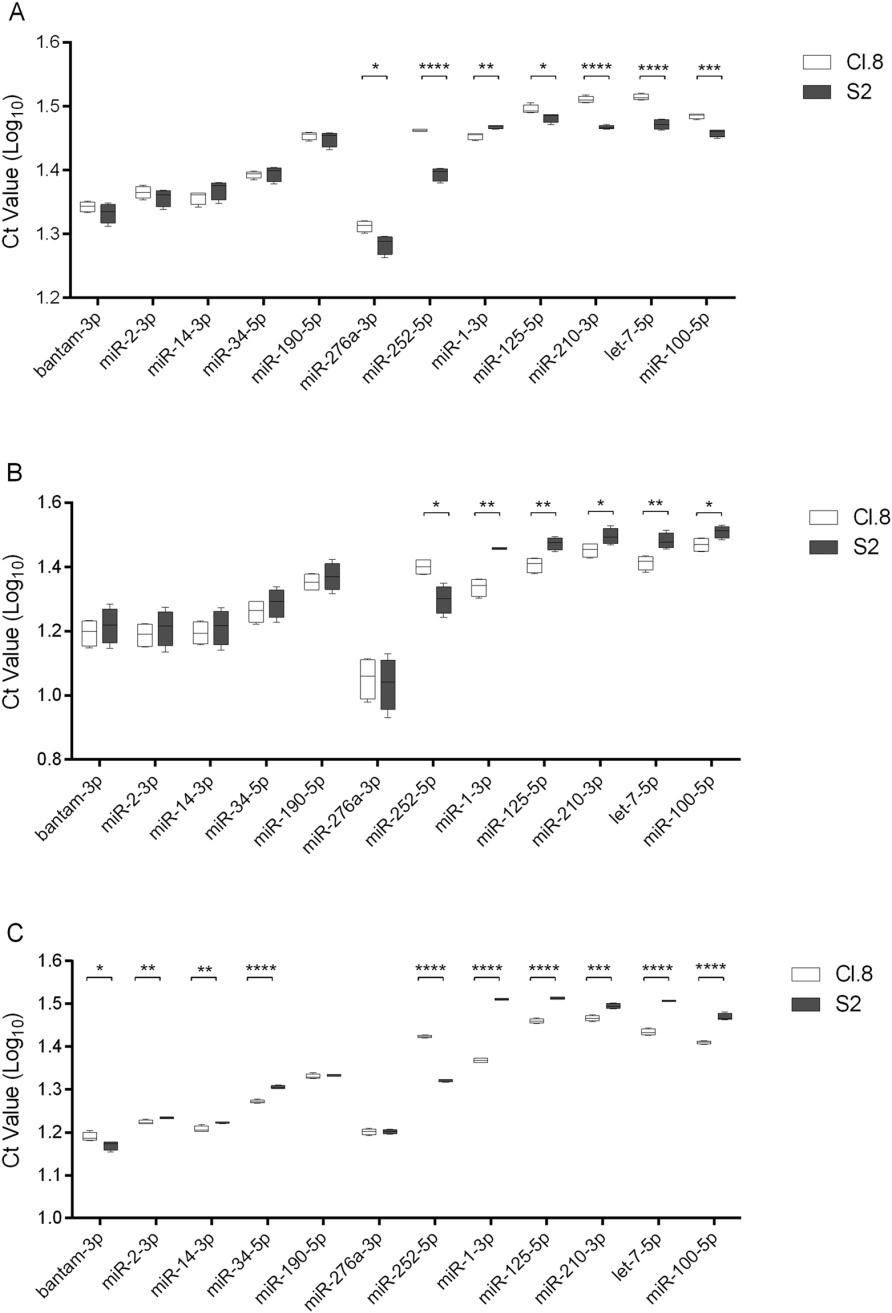
qRT-PCR data for microRNAs detected in extra- and intracellular fractions of Cl.8 and S2 cell lines. (**A**) Extracellular vesicles (EVs) and (**B**) extracellular Ago-1 containing immunoprecipitates (EAgo-1) were isolated from the cell-free conditioned media of Cl.8 and S2 cell cultures and different miRNAs were detected using qRT-PCR. (**C**) The intracellular expression levels of the selected miRNAs were measured as well. The data (log10 Ct values) are shown as box plots (min to max with the rectangle spanning the first to the third quartile including median) of four biological replicates, run in duplicate. Statistical analyses were performed with GraphPad Prism 6 (Graphpad Software Inc.). Significant differences (p < 0.05, p < 0.01, p < 0.001 and p < 0.0001) between the data obtained for specific miRNAs in the extracellular media of the two cell lines were found, after a log transformation, via a t-test (with or without two-sided Welch’s correction) and are indicated by (an) asterisk(s) (*, **, *** and **** respectively).

Two extracellular fractions were purified from the cell-free conditioned media of the selected cell lines, namely the EVs and EAgo-1, and subjected to immunoblot analysis to verify proper isolation (Fig. 1). Afterwards, the presence of twelve specific miRNAs was analysed via qRT-PCR: miR-1-3p; miR-2-3p; miR-14-3p; miR-34-5p; miR-100-5p; miR-125-5p; miR-190-5p; miR-210-3p; miR-252-5p; miR-276a-3p; bantam-3p; and let-7-5p. These miRNAs were selected from miRBase^7^ (Release 21.0) based on their known role in regulating several aspects of fruit fly development^44–46^. In addition, this pool of miRNAs contains both 5p- and 3p- miRNAs, which is of interest considering that not only 5p-miRNAs are functional as it was initially thought, but also 3p-miRNAs (*i.e*. derived from the 5′ and 3′ arm of the precursor, respectively)^47^. The twelve miRNAs revealed to be present in the analysed extracellular fractions, *i*.*e*. EVs and EAgo-1, purified from the extracellular media of both cell lines (Fig. 2A,B). Moreover it is interesting to note that no obvious biases towards the 5p- or 3p-type of miRNAs are observed (for example, miR-14-3p and miR-34-5p are more abundant while miR-210-3p and let-7-5p are less abundant).

Noteworthy, in order to allow comparisons of each fraction between both cell lines, Cl.8 and S2 cells were both seeded at the same density and each incubated until confluence was achieved before collecting the conditioned media. In addition, for cDNA synthesis, equal quantities of RNA were used for the EV and EAgo-1 fraction of both cell lines. Although all miRNAs are present in both extracellular fractions of the two cell lines, their abundance is variable, with significant differences between the cell lines. In fact, when compared to the data obtained from S2 cell cultures, six miRNAs are significantly less abundant in EVs derived from Cl.8 cell cultures, namely miR-100, miR-125, miR-210, miR-252, miR-276a and let-7, as shown by their higher Ct values. On the contrary, miR-1 is more abundant in Cl.8 EVs than in S2 EVs, *i*.*e*. lower Ct value. The presence of the remaining miRNAs in EVs does not differ significantly between the cell lines (Fig. 2A). On the other hand, the abundance of miR-1, miR-100, miR-125, miR-210 and let-7 is higher in the Cl.8 EAgo-1 fraction, while miR-252 is more abundant in EAgo-1 from S2 cells. The levels of the remaining EAgo-1 miRNAs do not differ between both cell lines (Fig. 2B).

Finally, it is interesting to note that the relative abundance of miR-100, miR-125, miR-210 and let-7 in extracellular fractions of S2 versus Cl.8 is inverted, as these miRNAs are more abundant in S2 EVs compared to Cl.8 EVs, although less abundant in S2 EAgo-1 compared to Cl.8 EAgo-1.

### MiRNA intracellular expression

Since the abundance of several miRNAs revealed to be significantly different between the Cl.8 and S2 extracellular fractions, the question whether these differences would correlate with differences in their intracellular expression remains to be answered. Therefore, the intracellular expression levels of these miRNAs were also measured (Fig. 2C). Interestingly, with the exception of miR-252 and miR-276a, all measured miRNAs exhibit a higher expression (*i*.*e*. lower Ct values) in Cl.8 cells. This is in accordance with the higher abundance of these miRNAs in Cl.8 derived EAgo-1 compared to S2 EAgo-1 (Fig. 2B). In this context, it is noteworthy that an inverted trend is observed in the EV fractions, since these miRNAs, except for miR-1, are more abundant in S2 EVs than in Cl.8 EVs (Fig. 2A).

On the other hand, lower expression of miR-252 in S2 versus Cl.8 cells is in line with its lower abundance in both S2 EVs and EAgo-1 (Fig. 2). In addition, although the intracellular expression levels of miR-276a do not differ between Cl.8 and S2 cells (Fig. 2C), this miRNA is remarkably more abundant in S2 EVs than in Cl.8 EVs (Fig. 2A). In conclusion, Table 1 offers a summary of the significant differences.

**Table 1:** Significantly differential abundance of *D*. *melanogaster* miRNAs when comparing the Cl.8 and S2 cell lines.

	Higher abundance in					
	EVs		EAgo-1		Intra	
bantam-3p	ns		ns		S2	(*)
miR-2-3p	ns		ns		Cl.8	(**)
miR-14-3p	ns		ns		Cl.8	(**)
miR-34-5p	ns		ns		Cl.8	(****)
miR-276a-3p	S2	(*)	ns		ns	
miR-252-5p	S2	(****)	S2	(*)	S2	(****)
miR-1-3p	Cl.8	(**)	Cl.8	(**)	Cl.8	(****)
miR-125-5p	S2	(*)	Cl.8	(**)	Cl.8	(****)
miR-210-3p	S2	(****)	Cl.8	(*)	Cl.8	(***)
let-7-5p	S2	(****)	Cl.8	(**)	Cl.8	(****)
miR-100-5p	S2	(***)	Cl.8	(*)	Cl.8	(****)

Significant differences (p) (*p < 0.05; **p < 0.01; ***p < 0.001; ****p < 0.0001). Abbreviations: EVs: extracellular vesicles; EAgo-1: extracellular Argonaute-1 containing immunoprecipitates; Intra: intracellular; ns: not significant.

Moreover, although the abundance of these miRNAs is not significantly different between the Cl.8 and S2 extracellular fractions, the intracellular expression levels of miR-2, miR-14 and miR-34 are higher in Cl.8 cells compared to S2 cells, while bantam is more expressed in S2 cells than in Cl.8 cells (Fig. 2C).

### Differential expression analysis of small RNA sequencing data

In a complementary approach, small RNA sequencing (RNA-seq) reads of S2 cells and derived EVs were analysed and compared with corresponding qRT-PCR results. Abundant miRNAs in S2 EVs and S2 cells (Fig. 2A,C), *i*.*e*. bantam, miR-14, miR-34 and miR-276a, also display a higher number of read counts in the RNA-seq data (Table 2). In addition, less abundant miRNAs in S2 cells and S2 EVs (miR-100, miR-125, miR-210 and let-7) are characterized by high Ct values (Fig. 2A,C) and very low read count numbers (Table 2). Taken together, these observations are in line with the qRT-PCR results. Here, it is important to note that small RNA-seq can discriminate miR-2-3p in three different subtypes (miR-2a-3p, miR-2b-3p and miR-2c-3p) which only differ 1 or 2 nucleotides (outside the seed region). Nevertheless, it is interesting that a certain subtype is more present (miR-2b-3p) than others. This might indicate that a certain miR-2-3p subtype might be more secreted or retained in the cell compared to other subtypes.

**Table 2:** Small RNA sequencing data are in agreement with qRT-PCR results (Fig. 2).

miRNA	S2 Cell			S2 EV			Log2FoldChange	p_adj_ value
dme-bantam-3p	9909	832	752	524	534	309	−3.07	0.042
dme-miR-2a-3p	8	18	11	6	11	17	−0.07	0.968
dme-miR-2b-3p	111	237	230	124	388	244	0.39	0.800
dme-miR-2c-3p	0	2	1	0	8	3	1.80	0.310
dme-miR-14-3p	1889	2519	1525	92	287	404	−2.92	0.024
dme-miR-34-5p	610	728	481	255	468	228	−0.94	0.434
dme-miR-190-5p	61	10	10	17	4	4	−1.70	0.275
dme-miR-276a-3p	513	1165	942	392	1946	1375	0.50	0.736
dme-miR-252-5p	0	9	10	2	10	7	0.02	0.987
dme-miR-1-3p	1	1	2	8	0	4	1.50	0.377
dme-miR-125-5p	NA	NA	NA	NA	NA	NA	NA	NA
dme-miR-210-3p	NA	NA	NA	NA	NA	NA	NA	NA
dme-let-7-5p	4	0	1	6	27	13	3.10	0.052
dme-miR-100-5p	0	0	1	0	6	6	3.24	0.077

Small RNA sequencing data of S2 cells and derived EVs, on three biological replicates, were analysed using edgeR. Normalised counts per sample of the twelve miRNAs studied with qRT-PCR and their corresponding adjusted p-values (α < 0.05). Abundant miRNAs (low Ct values in qRT-PCR) display high read counts, while less abundant miRNAs (high Ct values in qRT-PCR) are characterized by (very) low read counts. NA values indicate miRNAs which have no reads in at least four out of six samples. Log2FoldChange values reflect the differential expression between S2 cells and EVs. Positive and negative values indicate upregulated and downregulated miRNA abundances in EVs, respectively. Abbreviations: EV: extracellular vesicle; p_adj_: raw p-value adjustment.

**Figure 3:**
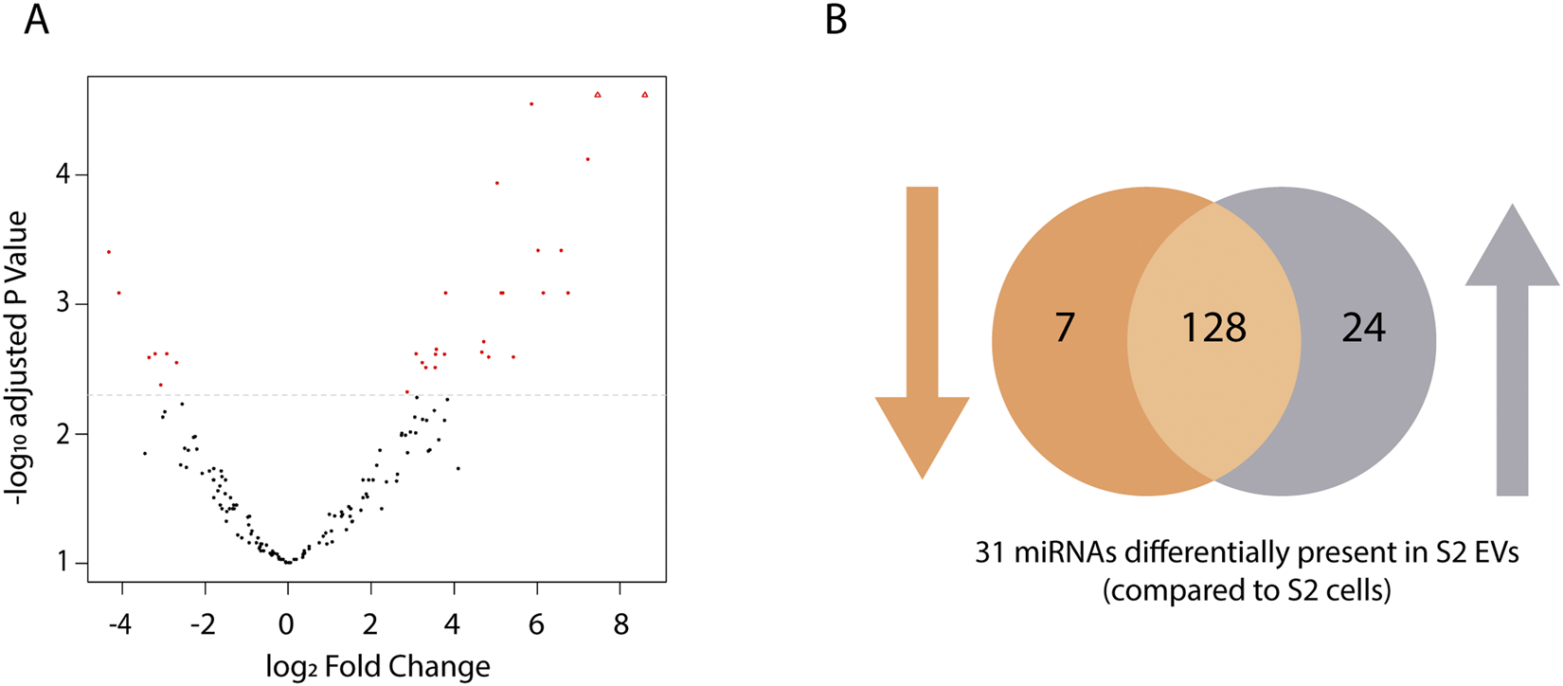
Differentially expressed miRNAs as measured in small RNA-seq data of S2 cells and derived EVs. Differential expression analysis was performed using edgeR on three biological replicates of both groups. MiRNAs were retained with a CPM above 1 in at least 3 samples. (**A**) Volcano plot indicating 31 significantly different miRNAs in S2 cells and EVs (red dots) and (**B**) the corresponding Venn diagram presenting the amount of miRNAs with increased (24) or decreased (7) abundance in S2 EVs compared to S2 cells. Abbreviations: EV: extracellular vesicle.

At last, based on the differential expression analysis, the abundances of 31 miRNAs significantly differed between S2 EVs and S2 cells (Fig. 3). Seven out of 31 miRNAs were significantly more abundant in S2 cells compared to S2 EVs, while the remaining 24 miRNAs were more abundant in S2 EVs (Table 3).

**Table 3:** Differentially expressed miRNAs.

miRNA	S2 Cell			S2 EV			Log2FoldChange	p_adj_ value
**A**								
dme-miR-9381-5p	7	16	2	319	1730	2406	7.46	0.000004
dme-miR-9378-3p	0	0	0	3	45	96	8.60	0.000192
dme-miR-9377-3p	1	7	1	67	171	262	5.87	0.000283
dme-miR-303-3p	1	0	0	2	65	119	7.22	0.000757
dme-miR-4958-5p	1	6	1	32	84	156	5.04	0.001156
dme-miR-2c-5p	0	0	0	24	1	11	6.58	0.003838
dme-miR-9369-5p	0	0	0	2	9	13	6.02	0.003838
dme-miR-4949-3p	1	0	0	86	1	2	6.15	0.008146
dme-miR-4962-3p	0	0	0	5	2	6	5.13	0.008146
dme-miR-4963-3p	1	3	1	36	26	24	3.80	0.008146
dme-miR-4970-5p	0	0	0	6	2	5	5.18	0.008146
dme-miR-9379-5p	0	0	0	38	1	2	6.75	0.008146
dme-miR-133-3p	0	0	0	3	2	4	4.72	0.019416
dme-miR-979-5p	2	2	0	11	14	32	3.57	0.022128
dme-miR-316-3p	1	0	0	27	2	3	4.67	0.023358
dme-miR-313-5p	4	7	2	36	26	52	3.08	0.024110
dme-miR-9380-3p	1	0	0	6	3	8	3.77	0.024288
dme-miR-990-3p	1	4	1	8	18	58	3.55	0.024288
dme-miR-9370-3p	1	0	0	56	1	3	4.83	0.025413
dme-miR-989-3p	2	1	0	163	2	0	5.43	0.025413
dme-miR-279-5p	2	20	3	38	78	121	3.23	0.028122
dme-miR-1016-3p	1	0	0	3	4	5	3.32	0.030763
dme-miR-960-5p	1	1	0	2	11	22	3.54	0.030763
dme-miR-289-5p	4	6	2	16	13	56	2.87	0.047273
**B**								
dme-miR-2a-2-5p	243	54	76	3	11	3	−4.32	0.004
dme-miR-34-3p	10	72	53	3	3	2	−4.07	0.008
dme-bantam-5p	2734	673	482	260	103	58	−3.21	0.024
dme-miR-14-3p	1889	2519	1525	92	287	404	−2.92	0.024
dme-miR-980-5p	10	99	83	3	11	3	−3.35	0.026
dme-miR-8-3p	2784	1054	861	244	267	216	−2.69	0.028
dme-bantam-3p	9909	832	752	524	534	309	−3.07	0.042

Normalised counts per sample of miRNAs with an increased (**A**) and decreased (**B**) abundance in S2 EVs. Log2FoldChange values reflect the differential expression between S2 cells and EVs. Positive and negative values indicate upregulated and downregulated miRNA abundances in EVs, respectively. MiRNAs were ranked in both tables (top to bottom) based on their adjusted p-values (α < 0.05). Abbreviations: EV: extracellular vesicle; p_adj_: raw p-value adjustment.

## Discussion

In this work, we have investigated the presence of ex-miRNAs in insect cell cultures. We have addressed this question by studying two *Drosophila* cell lines, namely Cl.8 and S2, which have different origins and properties. Our study shows the presence of miRNAs in the cell-free conditioned media of both cell lines. More specifically, we demonstrate that ex-miRNAs are present in two fractions, namely EVs and EAgo-1 (Fig. 2A,B). This suggests that ex-miRNAs are protected from degradation by either encapsulation in EVs or association with the stable Ago-1 protein. Likewise, studies in blood and plasma identified stable circulatory RNAs, suggesting their presence in an RNase-resistant form^24,48^. In accordance with our data, several studies in mammals have indicated the presence of miRNAs in EVs derived from human blood, cultured cells and virtually all other extracellular fluids, such as breast milk, semen, cerebrospinal fluid, urine and saliva^25,29–35^. Although in mammals EV-associated miRNA was originally thought to be the main extracellular form, two independent studies showed that most of the miRNAs in plasma, serum and conditioned media were EV-free but associated with proteins of the Ago family^23,36^. More specifically, human Ago-2 was clearly detected in plasma and associated with myriad miRNAs. A plausible explanation for stable Ago-associated miRNAs (Ago-miRNAs) in the harsh extracellular environment is that mature miRNAs naturally associate with them within the cell^13^ as part of the RISC and that Ago-miRNA complexes are highly nuclease/protease resistant, as they can remain stable in cell lysates for up to two months^16,23^. Our results clearly indicate that the presence of miRNAs in extracellular vesicles and Ago-containing ribonucleoprotein complexes (Fig. 2A,B) is a more widespread phenomenon that is not restricted to mammalian systems.

Each miRNA of the analysed group, which includes 5p- and 3p-miRNAs, was detected in both the EV and EAgo-1 fraction of both cell lines (Fig. 2). Likewise, it has been shown in mammals that certain miRNAs are present in both fractions, although others appear to be exclusively present in only one of them. For example, miR-100 and miR-210 (two miRNAs we clearly detect in both fractions) exclusively associate with extracellular Ago-2 in human plasma^36^. Here, it is important to note that only a very small set of miRNAs was tested in comparison to the referred studies. Thus, also in insects, it cannot be excluded that certain miRNAs exclusively associate with either EVs or EAgo-1.

In addition, small RNA-seq data of S2 cells and S2 EVs were included as a second approach to verify our qRT-PCR data. Small RNA-seq reads were aligned to all available mature *D*. *melanogaster* miRNAs on miRBase^7^ enabling the comparison of intracellular and extracellular reads of 469 miRNAs. The read counts of the twelve miRNAs under study are in line with our qRT-PCR data, since abundant miRNAs (low Ct values in qRT-PCR; Fig. 2) display higher read count numbers (Table 2) while less abundant miRNAs (high Ct values; Fig. 2) are characterized by low read count numbers (Table 2). Moreover, based on differential expression analysis, 31 miRNAs were identified with significantly different abundances between S2 cells and S2-derived EVs (Fig. 3). While seven out of 31 miRNAs were significantly more abundant in S2 cells compared to S2 EVs, the opposite is witnessed for the remaining 24 miRNAs (Table 3). Taken together, these observations point towards the controlled miRNA secretion in EVs, as certain miRNAs are retained more in the cells while others are relatively more abundant in EVs. In accordance with our data, multiple studies in mammals demonstrate controlled and selective miRNA secretion, as numerous miRNAs could not be detected in human plasma and conditioned media, although they are present intracellularly^23,37^.

Since the levels of certain miRNAs are significantly different between each extracellular fraction of both cell lines (Fig. 2A,B), we hypothesized that this might result from differences in their intracellular expression levels (Fig. 2C). Remarkably, although a higher intracellular expression seems to correlate with a higher abundance in the EAgo-1 fraction, an inverted trend is observed for the EV fraction (Table 1). In addition, even though the intracellular expression of miR-276a does not differ between S2 and Cl.8 cells, this miRNA is significantly more abundant in S2 EVs compared to EVs derived from Cl.8 culture medium. Taken together, these observations indicate that S2 derived EVs contain more copies of several miRNAs although their intracellular expression is lower compared to Cl.8 cells. In other words, our results support the occurrence of selective miRNA secretion and sorting in EVs. Interestingly, antiviral siRNAs have recently been demonstrated to be present in haemocyte-derived EVs in adult flies in order to potentiate a systemic antiviral response^49^. Likewise, EVs derived from haemocyte-like S2 cells might also be selectively loaded and able to mediate intercellular communication.

In recent years, studies of ex-miRNAs in mammalian systems accumulated, since these ex-miRNAs are secreted by cancer cells and might therefore be promising non-invasive biomarkers of tumorigenesis^28,50,51^. Nevertheless, whether ex-miRNAs might mediate intercellular communication remains debatable. For example, although protein complexes can be secreted, multiple studies suggest that extracellular Ago-miRNA complexes are by-products released during cell death and are able to circulate for a long time due to the stability of Ago proteins^23,26,28,52^. Likewise, it has been demonstrated *in vitro* that the miRNA content in culture media of mammalian cells coincides with cell death rate and *in vivo* studies indicate that damage of certain organs causes an increase of tissue-specific miRNAs in circulation^23,52–55^. Nevertheless, even though they would be released during cell death, the exact potential of the extracellular Ago-miRNA complexes remains unidentified. Our data show that insect EAgo-1 associated miRNAs are present in the (cell-free) conditioned media of *Drosophila* cell lines, but whether they are a result of non-selective release during cell death or selective secretion remains unknown. On the contrary, in mammalian biofluids, the situation is clearer for miRNAs encapsulated in EVs. EV sorting and secretion is controlled by the cell and specific EV-surface proteins enable targeted delivery^19^. In this context, several studies demonstrated the functional transfer of exosomal RNA to recipient cells^27,38^. Nevertheless, the question was also raised whether EVs contain miRNAs in physiologically relevant concentrations^19,26^. Thus, the hypothesis of ex-miRNAs in EVs or bound to EAgo-1 mediating intercellular communication remains to be further studied.

To conclude, we have shown the presence of ex-miRNAs in extracellular conditioned media of insect cells. To our knowledge, this is the first time ex-miRNAs are identified in different fractions (namely EVs and EAgo-1) of extracellular media of cultured *Drosophila* cells. Comparison of miRNA abundancies between both cell lines indicate selective miRNA sorting and/or secretion. Our findings open new avenues to study the potential role of ex-miRNAs in intercellular communication in insects, a so far underexplored, but conceptually very intriguing, research question.

## Methods

### Cl.8 and S2 cell cultures

The Clone 8 (Cl.8) cell line was maintained in Shields and Sang M3 Insect medium (Sigma-Aldrich), supplemented with 2% heat-inactivated Fetal Bovine Serum (FBS) (Sigma-Aldrich), 2,5% fly extract, 5 µg/ml human insulin (Sigma-Aldrich), 0.25 µg/ml Amphotericin B (Gibco, Life Technologies), 100 U/ml penicillin and 100 μg/ml streptomycin (Gibco, Life Technologies). The Schneider 2 (S2) cell line was also maintained in Shields and Sang M3 Insect medium supplemented with 1 g/l yeast extract (Sigma-Aldrich) and 2.5 g/l bactopeptone (BD Biosciences), 10% heat-inactivated FBS, 0.25 µg/ml Amphotericin B, 100 U/ml penicillin and 100 μg/ml streptomycin. The cells were subcultured weekly and maintained at 25 °C.

To study the presence of miRNAs in EVs or bound to EAgo-1, optimized cell culture media lacking vesicles and Ago proteins were required. EV-depleted FBS and fly extract were obtained by ultracentrifugation (110,000 g, 4 h, 4 °C). In addition, the fly extract was depleted of Ago proteins by using a 30 kDa MWCO Centriprep™ Centrifugal Filter Units (Millipore®).

### Isolation of extracellular vesicles and extracellular Argonaute-1 from conditioned media

Conditioned media were obtained by seeding Cl.8 and S2 cells (1.5 × 10^6^ cells/ml) in T-150 cell culture flasks using the optimized cell culture media. After reaching 80% confluency, the cells were collected by centrifugation (1000 g, 10 min, 4 °C) and stored in 100 µl PBS at −80 °C until further use. Supernatants (*e*.*g*. the conditioned media) were depleted from apoptotic bodies by centrifugation at 5000 g for 10 min at 4 °C and subsequently further concentrated using 30 kDa MWCO Centriprep™ Centrifugal Filter Units (Millipore®). Finally, each sample was divided in half for further processing, namely for EV and EAgo-1 isolation.

EVs were purified using part one of the exoRNeasy serum/plasma kit (Qiagen) according to the manufacturer’s protocol. In brief, each sample was mixed with an equal volume of XBP buffer and applied to the exoEasy spin column. After centrifugation, the bound EVs were washed with XWP washing buffer.

EAgo-1 was isolated by means of immunoprecipitation (IP). Briefly, 75 µl of Dynabeads M-280 Sheep Anti-Mouse IgG slurry (Thermo Fisher Scientific) were washed with washing buffer (PBS supplemented with 0.1% BSA and 2 mM EDTA) according to the manufacturer’s protocol and incubated with 1 µg of mouse monoclonal anti-Ago-1 antibody (gift from Prof. Haruhiko Siomi, Keio University School of Medicine, Japan) for 2 h at 4 °C. The pre-incubated beads and antibody were added to the sample and incubated overnight at 4 °C. Beads were washed three times with washing buffer and eluted in 250 µl of QIAzol Lysis Reagent (Qiagen) for RNA isolation or in 50 µl NuPAGE LDS Sample Buffer (4×) (Invitrogen^TM^) for immunoblotting.

Both the purified EV and EAgo-1 fraction were subjected to immunoblot analysis to verify proper isolation (Fig. 1). In addition, a mock IP experiment (Supplementary Fig. S2) was included as a negative control for EAgo-1 isolation.

### Immunoblotting

Protein concentrations of isolated Cl.8 and S2 EVs were determined by the bicinchoninic acid (BCA) assay. Equal protein amounts were analysed by SDS-PAGE using a NuPAGE 4-12% Bis-Tris gradient gel (Thermo Fisher Scientific) and subsequently electroblotted to polyvinylidene difluoride (PVDF) membranes (Thermo Fisher Scientific). The PVDF membrane was blocked in 5% low-fat dry milk powder in Tris-buffered saline (TBS) for 2 h at room temperature (RT) and subsequently incubated with primary antibodies overnight at 4 °C. The PVDF membrane was subsequently washed in TBS and then incubated with horseradish peroxidase (HRP)-conjugated goat anti-Mouse secondary antibody (Dako, Glostrup, Denmark) for 45 min at RT. Finally, the membrane was washed in Tris buffer and proteins were visualized using the SuperSignal^TM^ West Dura Extended Duration Substrate (Thermo Fisher Scientific) and imaged on the Chemidoc MP Imaging System (Bio-Rad). The primary antibodies used were Rho1 (DSHB Hybridoma Product p1D9 (anti-rho1), Parkhurst, S.), ROP (DSHB Hybridoma Product Rop 4F8, Rubin, G.M.) and Rab 11 (cat.no 610656, BD Biosciences), three common EV-marker proteins.

EAgo-1 immunoprecipitates were eluted in 50 µl NuPAGE LDS Sample Buffer (4×) (Invitrogen™) at 70 °C for 10 min. Afterwards, S2 and Cl.8 IP products were analysed by immunoblotting according to the above-mentioned protocol. The primary antibody used was anti-Ago-1 (gift from Prof. Haruhiko Siomi, Keio University School of Medicine, Japan), the secondary antibody HRP-conjugated goat anti-mouse (Dako, Glostrup, Denmark).

### RNA extraction and cDNA synthesis

RNA encapsulated in EVs was collected using the second part of the exoRNeasy serum/plasma kit, RNA from immunoprecipitated EAgo-1 was extracted with the miRNeasy Serum/Plasma Kit (Qiagen) and a separate miRNA-enriched fraction could be purified from the cellular fraction with the miRNeasy Mini kit and RNeasy MinElute Cleanup kit (Qiagen), all according to the manufacturer’s protocol. Quality and concentration of the resulting RNA samples were measured on a Nanodrop spectrophotometer (NanoPhotometer N60, Implen) and analysed using the Agilent Bioanalyzer Small RNA chip (Agilent Technologies, Inc.). Equal amounts of RNA (20 ng for EAgo-1 immunoprecipited RNA and 100 ng for EV encapsulated RNA) were used for cDNA synthesis using the qScript^TM^ microRNA cDNA Synthesis Kit (Quanta Bio) following the manufacturer’s protocol. This protocol includes polyadenylation of the miRNAs in a poly(A) polymerase reaction followed by cDNA synthesis using an oligo-dT adapter primer. This adapter primer allows amplification of cDNAs in quantitative real-time PCR (qRT-PCR) reactions due to a unique sequence at its 5′ end. The obtained cDNA was then diluted ten-fold with Milli-Q water (Millipore).

### Quantitative real-time PCR

Using a specific forward and a universal poly(T) adaptor reverse primer (Supplementary Table S1), amplification of cDNA samples was performed. Forward primers were designed to cover at least 80% of the miRNA sequence and random nt were added on the 5′ end to raise the melt temperature to 60 °C. Primer pairs were validated by designing relative standard curves for gene transcripts with serial (5×) dilutions of appropriate cDNA samples. The correlation coefficient and efficiency of the qRT-PCR reaction were measured (R² = 0.995 − 1; Eff% = 90–110%). All qRT-PCR reactions were performed in duplicate in 96-well plates on a StepOne Plus System (ABI Prism, Applied Biosystems) according to the Fast SYBR Green PCR Master Mix protocol. Each reaction contained 5 µl of PerfeCTa SYBR Green FastMix, ROX (Quanta Bio), 0.5 µl of each forward and reverse primer (10 µM), 1.5 µl of Milli-Q water and 2.5 µl of cDNA. A no template control reaction was always included. The following thermal cycling profile was used: 95 °C for 10 min, followed by 40 cycles of 95 °C, 15 s and 60 °C, 1 min. Then, a melt curve analysis was performed to confirm the specificity of the qRT-PCR reactions. In addition, the amplification products of three miRNAs were analysed using horizontal agarose gel electrophoresis (1.2% agarose gel containing GelRed^TM^, Biotium) and visualized using UV. A single band of the expected size for each transcript was observed, which was further cloned and sequenced (TOPO® TA Cloning Kit for sequencing, Invitrogen) to confirm amplicon specificity. MiR-276a, bantam and miR-100 were chosen since they respectively represent miRNAs with high, intermediate and low abundances.

### Small RNA sequencing data and analysis

Briefly, small RNA libraries of S2 cells and derived EVs were prepared using the TailorMix microRNA Sample Preparation Kit V2 (SeqMatic) according to the manufacturer’s protocol. These small RNA libraries were sequenced on the Illumina MiSeq® System with a single read length of 50 nt. Sequencing quality control was monitored via PhiX spike-ins (2%). Illumina adapters/primers were removed from the raw reads with BBDuk (version 37.33; Bushnell B.). Subsequently, the processed reads were aligned to the available *D*. *melanogaster* mature miRNAs from miRBase (release 21.0)^7^ using Bowtie 2 (Galaxy Version 2.3.4.1; 10.1186/gb-2009-10-3-r25) and mapped read counts were obtained using the Idxstats command of SAMtools (Galaxy Version 2.0.1; 10.1093/bioinformatics/btp352). Differential expression analysis was performed using the R (R Core Team; version 3.3.3) packages Sartools (version 1.6.1: 10.1371/journal.pone.0157022) and edgeR (version 3.16.5; 10.1093/bioinformatics/btp616) (Supplementary Table S2). MiRNAs were only retained if they displayed a counts-per-million (cpm) above 1 in at least 3 samples.

## Electronic supplementary material

Supplementary information

Supplementary Table S2

## References

[1] Lee RC, Feinbaum RL, Ambros V (1993). The C. elegans heterochronic gene lin-4 encodes small RNAs with antisense complementarity to lin-14. Cell.

[2] Berezikov E (2011). Evolution of microRNA diversity and regulation in animals. Nat. Rev. Genet..

[3] Gomez-Orte E, Belles X (2009). MicroRNA-dependent metamorphosis in hemimetabolan insects. Proc. Natl. Acad. Sci. USA.

[4] Caygill EE, Johnston LA (2008). Temporal Regulation of Metamorphic Processes in *Drosophila* by the let-7 and miR-125 Heterochronic MicroRNAs. Curr. Biol..

[5] Jayachandran B, Hussain M, Asgari S (2013). An insect trypsin-like serine protease as a target of microRNA: Utilization of microRNA mimics and inhibitors by oral feeding. Insect Biochem. Mol. Biol..

[6] Chawla G, Sokol NS (2011). MicroRNAs in *Drosophila* development. Int. Rev. Cell Mol. Biol..

[7] Griffiths-Jones S, Grocock RJ, van Dongen S, Bateman A, Enright A (2006). J. miRBase: microRNA sequences, targets and gene nomenclature. Nucleic Acids Res..

[8] Rodriguez A, Griffiths-Jones S, Ashurst JL, Bradley A (2004). Identification of mammalian microRNA host genes and transcription units. Genome Res..

[9] Denli AM, Tops BBJ, Plasterk RHA, Ketting RF, Hannon GJ (2004). Processing of primary microRNAs by the Microprocessor complex. Nature.

[10] Gregory RI (2004). The Microprocessor complex mediates the genesis of microRNAs. Nature.

[11] Lund E (2004). Nuclear Export of MicroRNA Precursors. Science (80-.)..

[12] Knight SW, Bass BL (2001). A Role for the RNase III Enzyme DCR-1 in RNA Interference and Germ Line Development in *Caenorhabditis elegans*. Science.

[13] Miyoshi K, Okada TN, Siomi H, Siomi MC (2009). Characterization of the miRNA-RISC loading complex and miRNA-RISC formed in the *Drosophila* miRNA pathway. Rna.

[14] Okamura K, Ishizuka A, Siomi H, Siomi MC (2004). Distinct roles for Argonaute proteins in small RNA-directed RNA cleavage pathways. Genes Dev..

[15] Ellwanger DC, Büttner FA, Mewes HW, Stümpflen V (2011). The sufficient minimal set of miRNA seed types. Bioinformatics.

[16] Vodovar N, Saleh MC (2012). Of Insects and Viruses: The Role of Small RNAs in Insect Defence. Adv. In Insect Phys..

[17] Wynant N, Santos D, Vanden Broeck J (2017). The evolution of animal Argonautes: evidence for the absence of antiviral AGO Argonautes in vertebrates. Sci. Rep..

[18] Etheridge A, Gomes CPC, Pereira RW, Galas D, Wang K (2013). The complexity, function, and applications of RNA in circulation. Front. Genet..

[19] Makarova JA, Shkurnikov MU, Turchinovich AA, Tonevitsky AG, Grigoriev AI (2015). Circulating MicroRNAs. Biochem..

[20] Javidi MA (2014). *et al*. Cell-free microRNAs as cancer biomarkers: the odyssey of miRNAs through body fluids. Med. Oncol..

[21] Sayed ASM, Xia K, Salma U, Yang T, Peng J (2014). Diagnosis, prognosis and therapeutic role of circulating miRNAs in cardiovascular diseases. Hear. Lung Circ..

[22] Kawano Y (2013). Plasma viral MicroRNA profiles reveal potential biomarkers for chronic active epstein-barr virus infection. J. Infect. Dis..

[23] Turchinovich A, Weiz L, Langheinz A, Burwinkel B (2011). Characterization of extracellular circulating microRNA. Nucleic Acids Res..

[24] Mitchell PS (2008). Circulating microRNAs as stable blood-based markers for cancer detection. Proc. Natl. Acad. Sci..

[25] Chen X (2008). Characterization of microRNAs in serum: A novel class of biomarkers for diagnosis of cancer and other diseases. Cell Res..

[26] Turchinovich A, Tonevitsky AG, Burwinkel B (2016). Extracellular miRNA: A Collision of Two Paradigms. Trends Biochem. Sci..

[27] Valadi H (2007). Exosome-mediated transfer of mRNAs and microRNAs is a novel mechanism of genetic exchange between cells. Nat. Cell Biol..

[28] Turchinovich A, Tonevitsky AG, Cho WC, Burwinkel B (2015). Check and mate to exosomal extracellular miRNA: new lesson from a new approach. Front. Mol. Biosci..

[29] McDonald, M. K., Capasso, K. E. & Ajit, S. K. Purification and microRNA Profiling of Exosomes Derived from Blood and Culture Media. *J*. *Vis*. *Exp*. 1–7, 10.3791/50294 (2013). 10.3791/50294 PMC3727427 23792786

[30] Zhou Q (2011). Immune-related microRNAs are abundant in breast milk exosomes. Int. J. Biol. Sci..

[31] Vojtech L (2014). Exosomes in human semen carry a distinctive repertoire of small non-coding RNAs with potential regulatory functions. Nucleic Acids Res..

[32] Zhang Q (2013). Selective secretion of microRNA in CNS system. Protein Cell.

[33] El-Khoury V, Pierson S, Kaoma T, Bernardin F, Berchem G (2016). Assessing cellular and circulating miRNA recovery: the impact of the RNA isolation method and the quantity of input material. Sci. Rep..

[34] Delić D (2016). Urinary exosomal miRNA signature in type II diabetic nephropathy patients. PLoS One.

[35] Amanda M (2010). Exosomes from Human Saliva as a Source of microRNA Biomarkers. Oral Dis..

[36] Arroyo JD (2011). Argonaute2 complexes carry a population of circulating microRNAs independent of vesicles in human plasma. Proc. Natl. Acad. Sci..

[37] Shurtleff MJ, Temoche-Diaz MM, Karfilis KV, Ri S, Schekman R (2016). Y-box protein 1 is required to sort microRNAs into exosomes in cells and in a cell-free reaction. Elife.

[38] Lotvall J, Valadi H (2007). Cell to cell signalling via exosomes through esRNA. Cell Adh. Migr..

[39] Guo Xiangqian, Su Songkun, Skogerboe Geir, Dai Shuanjin, Li Wenfeng, Li Zhiguo, Liu Fang, Ni Ruifeng, Guo Yu, Chen Shenglu, Zhang Shaowu, Chen Runsheng (2013). Recipe for a Busy Bee: MicroRNAs in Honey Bee Caste Determination. PLoS ONE.

[40] Schneider I (1972). Cell lines derived from late embryonic stages of *Drosophila melanogaster*. J. Embryol. Exp. Morphol..

[41] Saleh M-C (2006). The endocytic pathway mediates cell entry of dsRNA to induce RNAi silencing. Nat. Cell Biol..

[42] Peel David J., Milner Martin J. (1990). The diversity of cell morphology in cloned cell lines derived from Drosophila imaginal discs. Roux's Archives of Developmental Biology.

[43] Lum L (2003). Identification of Hedgehog Pathway Components by RNAi in *Drosophila* Cultured Cells. Science (80-.)..

[44] Aravin A, Lagos-Quintana M, Yalcin A (2003). The Small RNA Profile during *Drosophila melanogaster* Development. Dev. Cell.

[45] Thompson BJ, Cohen SM (2006). The Hippo Pathway Regulates the bantam microRNA to Control Cell Proliferation and Apoptosis in *Drosophila*. Cell.

[46] Waldron JA, Newbury SF (2012). The roles of microRNAs in wing imaginal disc development in *Drosophila*. Biochem. Soc. Trans..

[47] Yang J-S (2011). Widespread regulatory activity of vertebrate microRNA* species. Rna.

[48] Tsui NBY, Ng EKO, Lo YMD (2002). Stability of endogenous and added RNA in blood specimens, serum, and plasma. Clin. Chem..

[49] Tassetto M, Kunitomi M, Andino R (2017). Circulating Immune Cells Mediate a Systemic RNAi-Based Adaptive Antiviral Response in *Drosophila*. Cell.

[50] Rabinowits G, Gerçel-Taylor C, Day JM, Taylor DD, Kloecker GH (2009). Exosomal microRNA: A diagnostic marker for lung cancer. Clin. Lung Cancer.

[51] Taylor DD, Gercel-Taylor C (2008). MicroRNA signatures of tumor-derived exosomes as diagnostic biomarkers of ovarian cancer. Gynecol. Oncol..

[52] Makarova JA (2016). Intracellular and extracellular microRNA: An update on localization and biological role. Prog. Histochem. Cytochem..

[53] Akat KM (2014). Comparative RNA-sequencing analysis of myocardial and circulating small RNAs in human heart failure and their utility as biomarkers. Proc. Natl. Acad. Sci..

[54] Laterza OF (2009). Plasma microRNAs as sensitive and specific biomarkers of tissue injury. Clin. Chem..

[55] Corsten MF (2010). Circulating MicroRNA-208b and MicroRNA-499 reflect myocardial damage in cardiovascular disease. Circ. Cardiovasc. Genet..

