# Supplementary material for: The presence of extracellular microRNAs in the media of cultured Drosophila cells
Source: Sci Rep. 2018 Nov 23;8:17312. doi: 10.1038/s41598-018-35531-z (PMC6251921; doi:10.1038/s41598-018-35531-z)
Supplement: Supplementary file 1 — Supplementary information [file 41598_2018_35531_MOESM1_ESM.pdf]

The presence of extracellular microRNAs in the media of cultured *Drosophila* cells

Stijn Van den Brande[1, ♦], Marijke Gijbels[1, ♦], Niels Wynant[1], Dulce Santos[1], Lina Mingels[1], Yannick Gansemans[2], Filip Van Nieuwerburgh[2], Jozef Vanden Broeck[1,*]

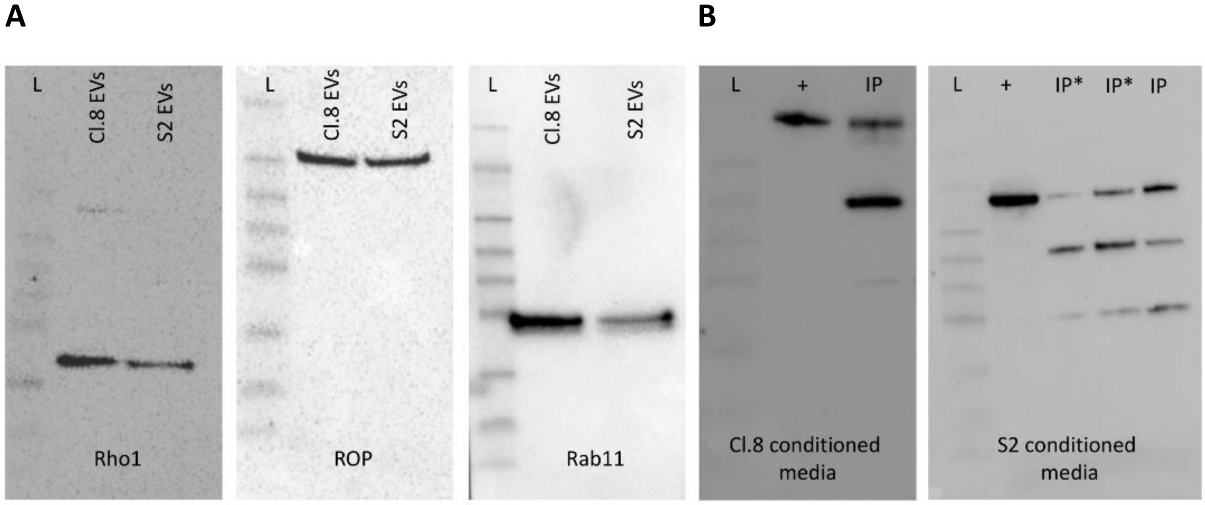

Supplementary Fig. S1. **Immunoblot analysis on EVs and EAgo-1 immunoprecipitates.** (**A**) EVs of both cell lines were subjected to immunoblot analysis for three common EV markers, which were clearly detected in the EV fractions purified from the extracellular media of both cell lines. (**B**) EAgo-1 IP was performed on the conditioned S2 and Cl.8 media as described in Methods section, but eluted in lithium dodecyl sulfate (LDS) instead of Qiazol. After elution, S2 and Cl.8 IP products were subjected to immunoblot analysis for EAgo-1, which was clearly detected in both IP products. Full-length blots of Fig. 1. Abbreviations: L: Seeblue Prestained 2 Protein Standard (Thermo Fisher); + : positive control sample (in this case, a protein extract of respectively Cl.8 and S2 cells); IP: Detection of EAgo-1 after immunoprecipitation. Both in the Cl.8 and S2 samples, we detect two additional bands at approximately 55 and 29 kDa, representing co-eluted antibody chains (heavy and light). IP*: Elution performed using Glycine buffer at different concentrations as a pilot experiment.

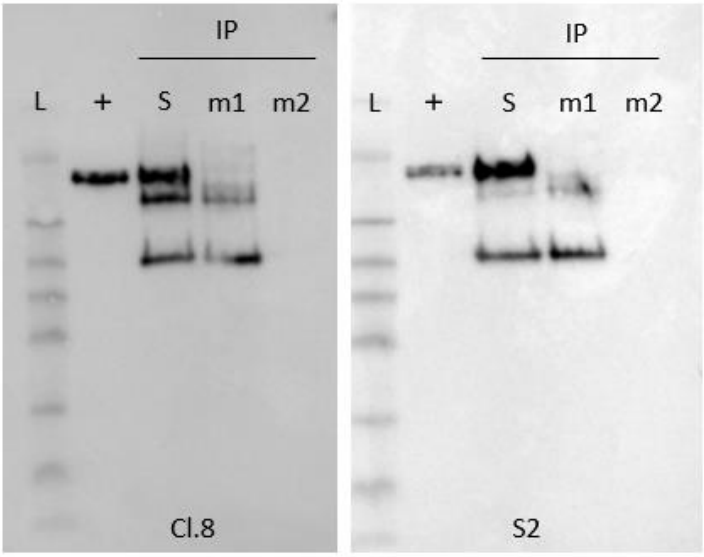

Supplementary Fig. S2. **Mock IP controls.** Two mock IPs were performed as negative controls to verify specific isolation of EAgo1. In mock 1 (m1), fresh cell culture media was added to the beads instead of the conditioned media (S). In the second mock (m2), no primary antibody was used during the IP protocol. EAgo-1 IP (S) and mock IPs (m1 & m2) were performed as described in Methods section, but eluted in lithium dodecyl sulfate (LDS) instead of Qiazol. After elution, the IP products were subjected to immunoblot analysis for Ago-1, which was clearly detected in the positive control and in the EAgo-1 IP (S) products, but not in the mock IPs eluates. In m1 eluate, only co-eluted antibody bands are seen; in m2 eluate, no bands are seen. Abbreviations: L: Seeblue Prestained 2 Protein Standard (Thermo Fisher); + : positive control sample (*i.e.* a protein extract of respectively Cl.8 and S2 cells); IP: Immunoprecipitation; S: sample (conditioned media); m1: mock 1 control; m2: mock 2 control.

Supplementary Table S1: **Oligonucleotide sequences of primers used for qRT-PCR.** The forward (F-) primer is a *D. melanogaster* miRNA specific primer, the reverse primer a universal one with sequence 5’- GCATAGACCTGAATGGCGGTA -3’. Accession numbers are included (miRBase Release 21.0).

**MiRNAs**	**F-primer**	**Accession number**
miR-1-3p	5’- CGCGGCTGGAATGTAAAGAAGTATGG -3’	MIMAT0000105
miR-2-3p	5’- CGCCTAGTATCACAGCCAGCTT -3’	MIMAT0000106, MIMAT0000107, MIMAT0000411
miR-14-3p	5’- AGGGCGGTCAGTCTTTTTCTCTC -3’	MIMAT0000120
miR-34-5p	5’- AGTGGCAGTGTGGTTAGCTGG -3’	MIMAT0000350
miR-100-5p	5’- GGCGAACCCGTAAATCCGAACTTG -3’	MIMAT0000357
miR-125-5p	5’- CGTCGCTTCCCTGAGACCCTAAC -3’	MIMAT0000397
miR-190-5p	5’- GCGGGGCAGATATGTTTGATATTCTTGG -3’	MIMAT0005467
miR-210-3p	5’- GTGCGTGTGACAGCGGCTAT -3’	MIMAT0000355
miR-252-5p	5’- GCCTAAGTACTAGTGCCGCAGG -3’	MIMAT0005516
miR-276a-3p	5’- GCGTAGGAACTTCATACCGTGCT -3’	MIMAT0000337
bantam-3p	5’- CGGGCAGTGAGATCATTTTGAAAGC -3’	MIMAT0020823
let-7-5p	5’- GCGGCTGAGGTAGTAGGTTGT -3’	MIMAT0000396

Supplementary Table S2: **S2 small RNA sequencing data.** Small RNA sequencing was executed on 3 biological replicates of S2 cells and EVs. Differential expression analysis was performed using edgeR. Amount of nucleotides (nt) and raw reads are included in the table.
